# How Does Perceived Support for Innovation Lead to Deviant Innovation Behavior of Knowledge Workers? A Moderated Mediation Framework

**DOI:** 10.3389/fpsyg.2022.890999

**Published:** 2022-05-31

**Authors:** Shujie Yuan, Xuan Liu

**Affiliations:** ^1^Department of Psychology, Huangshan University, Huangshan, China; ^2^School of Economics and Management, Nanjing Tech University, Nanjing, China

**Keywords:** perceived support for innovation, deviant innovation, innovation commitment, threatened self-identity, relationship

## Abstract

Many studies concerning deviant innovation behavior mainly focus on the influence of personality differences or leadership styles, and there is a lack of attention given to internal cognitive factors related to actors. Therefore, the purpose of this paper is to examine the internal mechanism of perceived support for innovation on deviant innovation behavior. A two-wave study was conducted among 393 knowledge workers from 10 knowledge-intensive enterprises in the People's Republic of China. Model 4 and Model 14 from SPSS macro PROCESS are used to test the mediating effect of innovation commitment and the moderating effect of threatened self-identity, respectively. The findings suggest that perceived support for innovation can significantly predict deviant innovation behavior; innovation commitment fully mediates the relationship between perceived support for innovation and deviant innovation behavior; public threat to self-identity plays a moderating role in the relationship between innovation commitment and deviant innovation behavior; and public threat to self-identity moderates the mediating effect of innovation commitment on perceived support for innovation and deviant innovation behavior. This study enriches the research on antecedent variables of deviant innovation behavior, and highlights the important role of situational factors on the whole mechanism.

## Introduction

The dynamic competition market and trade frictions of large countries have posed a great challenge to the adaptability of local enterprises. An increasing number of entrepreneurs realize that the key to enterprises enhancing their core competitiveness is to stimulate employee innovation. In knowledge-intensive enterprises that value innovation, knowledge workers have now become the object of strong organizational support, but compared to the methods and process of innovation, the enterprise attaches more importance to the results of innovation (Neumeyer et al., 2019). The common inference is that “innovation should be under the direct control of management” (Augsdorfer, 1996). However, resources are sometimes limited in the process of realistic innovation practice (Mainemelis, 2010), and employees cannot achieve their own innovation goals through formal channels and may turn to informal way-deviant behavior (Zhang and Tu, 2022), which is called “deviant innovation behavior”. It is characterized by bootlegging or underground innovation (Knight, 1967). Different from deviant employee behaviors such as lying, stealing, corruption, etc., which are generally considered to be avoidable due to losses caused, deviant innovation behavior often has altruistic motives and functional roles (Cheng, 2019). In the workplace, when a conflict between an employee's creativity and organizational authority or system will or may occur, if the individual insists that his or her creativity is conducive to the organization, he or she should choose to continue to practice this idea through unconventional means (Wang et al., 2018). Some studies have shown that individual variables such as overqualification, job characteristics such as remote position, and relationship status such as supervisor-subordinate task conflicts are closely related to deviant innovation behavior (Wang et al., 2018; Wang, 2019; Xiao, 2020). However, the psychological conflicts and cognitive changes in employees themselves were ignored (Helene and Philip, 2019). When employees receive much support for innovation from the organization, what are the characteristics of internal psychological changes and why would they want to disregard the rules to be observed and bootleg?

According to social exchange theory, there may be reciprocity and commitment between individuals and organizations when they gain value recognition and high trust (Eisenberger et al., 2001). Commitment often leads to target behavior and to deviant innovation behavior (Yuan and Liu, 2021). Perhaps perceived support for innovation influences deviant innovation behavior via innovation commitment. Meanwhile, when the idea for an innovation and the conventional mode are quite different or high responsibility requirements lead to innovation anxiety (Anwar and Niode, 2017), employees feel threatened by their self-identity. Threatened self-identity mainly refers to the immediate negative self-perception formed by an individual in a specific situation (Murtagh et al., 2012). Influenced by the psychological conflict of threats to self-identity, employees are more likely to behave in their prescribed roles to keep self-congruity. Therefore, threatened self-identity should be regarded as a conditional variable when exploring the influencing mechanism of the effect of perceived support for innovation on deviant innovation behavior.

Given the above, this study has an objective to analyze the internal mechanism of perceived support for innovation on deviant innovation behavior via the mediating effect of innovation commitment and the moderating effect of threatened self-identity. To conduct the study, we used Wenjuanxing, an online crowdsourcing platform in mainland China that provides functions equivalent to Amazon Mechanical Turk, to collect knowledge workers' perceptions about the studied variables. After this current introduction, the theoretical framework is developed. Then, the 6 hypotheses to be tested are presented and justified, followed by an explanation of the research model. Next, the analysis of the collected data is presented, followed by a discussion of the results and the main conclusions of the study.

## Theory and Hypotheses

Eisenberger et al. (2001) proposed the concept of perceived organizational support and regarded it as a comprehensive perception of how organizations evaluate employees' contributions and whether organizations are concerned about their wellbeing during the work process. This perceived support was proven to play an important role in stimulating social exchange between employees and organizations and enhancing the sense of obligation to achieve organizational goals (Rhoades and Eisenberger, 2002). Compared with perceived organizational support, perceived support for innovation refers to the subjective perception of organizational support for employees' pursuit and implementation of new ideas at work, which is more closely related to the target, i.e., creative behavior (Xu et al., 2021). Perceived support for innovation has an impact on employees' creative behavior, which, in turn, indirectly impacts their creative execution behavior (Gu et al., 2014a). A strong sense of support for innovation can create an advantageous psychological atmosphere for individuals who mobilize them to produce more positive emotions (Ding et al., 2018). In such an environment, knowledge workers often feel more confident about their innovative ideas and can become more creative as they experience positive emotions. Supportive external resources can be transformed into internal psychological advantages through cognitive evaluation, which can increase the sense of self-efficacy and even lead to self-expansion and the neglect of work boundaries, which can facilitate deviant innovation behavior (Gao et al., 2020; Ma and Guo, 2020). In addition, perceived support for innovation can stimulate employees' achievement motivation and positively impact their autonomous behavior (Lin, 2020). When knowledge workers perceive encouragement and support for innovation from their organization, they tend to increase their internal psychological resources, challenge conventions, and show high levels of creativity (Gu et al., 2014b). Thus, considering that deviant innovation behavior may be influenced by perceived support for innovation, we propose the first hypothesis:

**H_1_**: Perceived support for innovation has a positive effect on deviant innovation behavior.

Perceived support for innovation refers to employees' positive awareness of the openness of the organization. Based on the principle of reciprocity in social exchange, employees tend to engage in active thinking and have a strong sense of innovation. According to social exchange theory, employees are willing to make commitments and act in more ways that are beneficial to the organization because of the need to be recognized for their values (Settoon et al., 1996). Innovation commitment is a subordinate concept of commitment that emphasizes that the content of individual commitment is innovation rather than other types (Yuan and Liu, 2021). Highly committed employees tend to be more innovative than other employees because they consider their work to be self-fulfilling and are willing to show more talent and innovation in their work (Chen and Francesco, 2003). As a form of individual inner attachment to innovation, innovation commitment reflects not only employees' own behavior of giving back to the organization but also their high expectations for the realization of innovation goals or innovation performance (Yuan and Liu, 2021). The supportive and caring behavior of organizations and superiors makes it easier for employees to generate or pursue novel ideas, activities or relationships; helps them actively build lasting personal resources, such as problem-solving skills, and acquire new knowledge; and further enhances their beliefs about expectations for the success of innovation (Yang et al., 2008). Commitment reflects the degree to which an individual identifies with and participates in an organization (Yuan and Liu, 2021). Individuals have a sense of obligation to work, and in terms of job innovation, they also appreciate innovative behaviors through innovative self-efficacy (Xu and Zhao, 2020). Innovation commitment can influence the choice of innovation mode and plays a mediating role in the relationship between innovation climate and innovation performance (Wang and Ge, 2016). Overall, employees' perceived support for innovation should promote innovative behaviors through an inner sense of commitment and increase the probability of deviant innovation behavior. Thus, the second, third and fourth hypotheses to be tested are as follows:

H_2_: Perceived support for innovation has a positive effect on innovation commitment.
H_3_: Innovation commitment has a positive effect on deviant innovation behavior.
H_4_: Innovation commitment mediates the relationship between perceived support for innovation and deviant innovation behavior.

Deviant innovation behavior is regarded as an extrarole behavior of employees when their innovative ideas conflict with the rules of the organization and their superiors. Such a conflict may be caused by individuals' high sense of being overqualified in the workplace, which leads to paradoxical thinking and ultimately to these behaviors (Wang, 2019). This paradoxical thinking involves self-concept (Nanyangwe et al., 2021). Self-threat is a psychological state that measures the destruction of self-concept (Franzoi, 1982). This system of internal and external balance can be divided into the private self and public self. Private self-concepts are aspects of self-identity or self-concern that are difficult for others to understand, while public self-reflections are aspects of social identity or public display that are easy for others to discern (Franzoi, 1982). Knowledge workers have individualistic tendencies and a strong sense of freedom, do no follow authority, have high expectations and high goals and are willing to invest more resources to meet challenges (Parry and Urwin, 2011). In China, people are sometimes constrained by the ideology of “being superior to others and being inferior to others”, so they dare not go against the organization's requirements in public (Liu, 2019). When individuals fail in some innovation tasks, their innovative views are not supported and recognized by superiors and others, and the self-information that the individual usually receives from the outside world is negative. This sense of self-uncertainty often brings an experience of conflict for individuals, thus leading to psychological pressure. Such experience reduces the possibility of extrarole behavior to reduce the sense of self-threat. Individuals with a high sense of self-uncertainty are more willing to categorize and deindividuate (Hogg, 2014). In this way, conflict experiences lead to uncertainty in self-concept, and individuals increase in-role behavior while decreasing extrarole behavior. The level of threatened self-identity should influence the relationship between innovation commitment and deviant innovation behavior. Hence, we propose the fifth hypothesis to be tested in this study:

H_5_: Public threat to self-identity has a negative moderating effect on the relationship between innovation commitment and deviant innovation behavior. Private threat to self-identity has a negative moderating effect on the relationship between innovation commitment and deviant innovation behavior.

As mentioned above, the mediating effect of innovation commitment on the relationship between perceived support for innovation and deviant innovation behavior may also be moderated by threatened self-identity. In other words, when the level of public or private threat to self-identity is high, the indirect effect of perceived innovation support on deviant innovation behavior through the mediating role of innovation commitment is relatively weak. In contrast, when the level of public or private threat to self-identity is low, the indirect effect of perceived innovation support through innovation commitment on deviant innovation behavior is correspondingly enhanced. Thus, we consider it interesting to formulate the following hypothesis:

H_6_: Public threat to self-identity has a negative moderating effect on the mediating effect of innovation commitment. Private threat to self-identity has a negative moderating effect on the mediating effect of innovation commitment.

**Figure 1:**
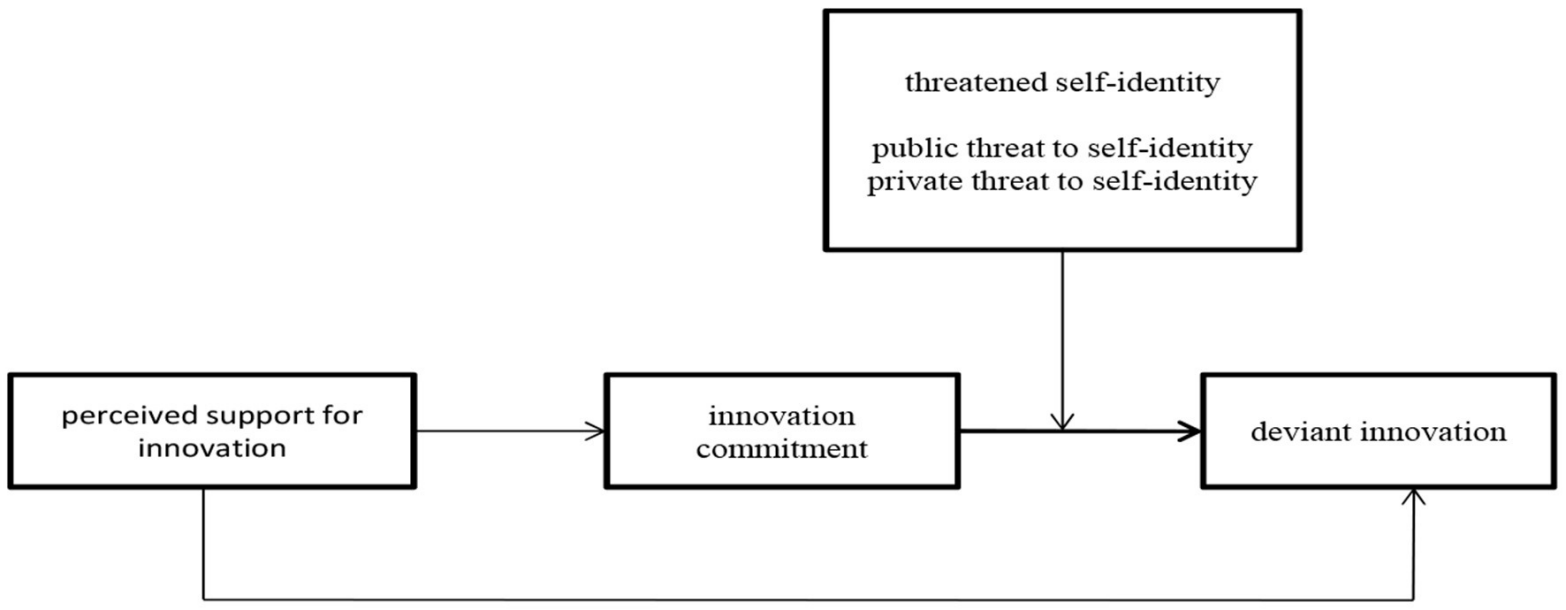
Proposed research model. Source: own elaboration.

Assuming that threatened self-identity moderates the relationship between innovation commitment and deviant innovation behavior, threatened self-identity is also likely to conditionally influence the strength of the indirect effect of perceived support for innovation on deviant innovation behavior through innovation commitment. This pattern of moderated mediation between the variables is depicted in Figure 1.

## Data Analysis

### Sample and Procedures

This study was a two-wave design conducted in 10 knowledge-intensive companies in the Yangtze River Delta of China from March through April 2021. These companies are mainly involved in software development, information technology and manufacturing. All workers were informed of the study via a WeChat working group and then received an invitation that described the aims, risks, benefits and process of the study, emphasized confidentiality, pointed out requirements for participating, and provided a link to the survey. A total of 456 knowledge workers consented to participate in the first survey. At time 1, the data of perceived support for innovation, threatened self-identity, and innovation commitment were collected. We received 432 valid responses for a 94.74% response rate. One month later, at time 2, a deviant innovation behavior scale was administered, and the second survey was answered by 393 out of the 432 initial respondents, for a 90.97% response rate. Among them, 55% were male, while 45% were female. In addition, 58.21% had bachelor's degrees, 28.1% had master's degrees, 7.6% had doctoral degrees, and 6.09% had an education below the bachelor's level. Given the nature of their job, 43.3% were core, and 56.7% were general. Regarding their positions, 69% were in intermediate positions or lower, and 31% were at associate senior positions or higher. The average age was 37.96 years (SD = 8.68).

### Measures

A Chinese version of all the measures based on the original English language scales was created using the translation and back-translation procedure (Brislin, 1986). The subjects were asked to respond to the survey using a five-point Likert scale (*1* = *strongly disagree, 5* = *strongly agree*). We measured deviant innovation behavior with nine items adopted from Lin et al. (2016). A sample item was “Although my superiors do not agree with my new plan, I will still go ahead with it”. Cronbach's α for this scale was 0.949. We assessed perceived support for innovation with eight items adopted from Siegel and Kaemmerer (1978). A sample item was “Our ability to work creatively is valued by our leader”. Cronbach's α for this scale was 0.944. According to the innovation theme, we adopted a five-item scale from Klein et al. (2001) that was adjusted appropriately for innovation commitment. A sample item was “I care a lot about whether I can achieve my innovation goals”. Cronbach's α for this scale was 0.918. We adopted a nine-item scale for threatened self-identity from Campbell and Sedikides (1999). There were four items for private threats to self-identity, such as “After rejecting my proposal or idea, I would feel a kind of inexplicable depression in my heart”. There were five items for public threats to self-identity, such as “Rejecting my proposal or idea will affect my image in front of other colleagues”. Cronbach's α was 0.887 for the former and 0.912 for the latter. Similar to previous research (Dewett, 2007; Jiang, 2018), we controlled for the employees' gender, education level, age, position and job nature.

## Analysis of the Results

To assess the potential influence of common method bias, we used Harman's one-factor test (Podsakoff et al., 2003). Four factors that accounted for 74.62% of the variance were extracted, and the first factor accounted for 31.57%. These findings demonstrate that common method bias is unlikely to be a significant problem in this study. Moreover, we tested for common method bias with a single-factor measurement model by combining all items into a single factor (Dedahanov et al., 2016). The findings indicate a poor model fit: comparative fit index *(CFI)* = 0.282; Tucker–Lewis index *(TLI)* = 0.220; standardized residual mean root *(SRMR)* = 0.262; χ^2^*/df* = 22.868; and root mean square error of approximation *(RMSEA)* = 0.236. These findings also demonstrate that common method bias is unlikely to be a significant issue in our study.

The discriminative validity of each scale was tested, and we found that the five-factor model was superior to the other models (χ^2^ = 731.813, *df* = 289, χ^2^*/df* = 2.532, *RMSEA* = 0.062, *CFI* = 0.951, *TLI* = 0.945, *SRMR* = 0.053). These findings demonstrate that there is good discriminative validity among the factors (Wen et al., 2018). In addition, the *CR* values of innovation commitment, private threat to self-identity, public threat to self-identity, perceived support for innovation and deviant innovation behavior were 0.920, 0.890, 0.914, 0.944 and 0.951, respectively (all > 0.7). The average variance extracted *(AVE)* values were 0.698, 0.731, 0.780, 0.740 and 0.686, respectively (all > 0.5 and all greater than the squared value of the correlation coefficient between the factors). Therefore, each factor had good construct reliability and convergence validity. Table 1 reports the means, standard deviations and bivariate correlations of all variables. As shown in Table 1, our results showed significant correlations between the dependent and independent variables and limited collinearity between our independent variables.

**Table 1:** Descriptive statistics and correlations (*N* = 393).

**Variables**	**Means**	**SD**	**1**	**2**	**3**	**4**	**5**	**6**	**7**	**8**	**9**
1. Perceived support for innovation	3.56	0.68									
2. Innovation commitment	3.69	0.64	0.35^**^								
3. Deviant innovation behavior	3.05	0.76	0.16^**^	0.40^**^							
4. Private threat to self-identity	2.60	0.77	−0.27^**^	−0.08	0.02						
5. Public threat to self-identity	2.46	0.85	−0.28^**^	−0.13^**^	0.04	0.71^**^					
6. Gender	0.55	0.50	−0.07	−0.08	−0.21^**^	−0.04	−0.09				
7. Age	37.91	8.67	−0.03	0.11^*^	0.16^**^	0.12^**^	0.15^**^	−0.14^**^			
8. Education	2.39	0.83	0.05	0.18^**^	−0.02	0.05	0.00	0.00	−0.03		
9. Position	2.55	1.23	−0.08	0.21^**^	0.20^**^	0.15^**^	0.14^**^	−0.13^**^	0.54^**^	0.31^**^	
10. Job nature	1.43	0.50	−0.02	0.01	0.08	0.17^**^	0.14^**^	−0.02	0.24^**^	0.09	0.10

* *p < 0.05*,

** *p < 0.01*,

^***^p < 0.001.

Following Preacher et al. (2010), we tested a path model specifying the indirect effects of perceived support for innovation on deviant innovation behavior through innovation commitment (X → M → Y). In addition, gender, age, education level, position and job nature were included as control variables. The purpose of this analysis was to test the significance of the direct and indirect effects from X to Y through M.

As shown in Table 2, the path model results showed that perceived support for innovation was positively related to deviant innovation (γ = 0.18, *p* < 0.01); thus, H1 was supported. Furthermore, as H2 proposed, perceived support for innovation was proven to be positively related to innovation commitment (γ = 0.32, *p* < 0.001) and thus supported H2. Similarly, the results showed that innovation commitment was positively related to deviant innovation behavior (γ = 0.47, *p* < 0.001), which supported H3. To test the mediating effect proposed by H4, we used a parametric bootstrap procedure with 20,000 Monte Carlo replications to estimate a confidence interval (*CI*) around the indirect effects (Preacher et al., 2010). The results showed a positive indirect effect of perceived support for innovation on deviant innovation behavior via innovation commitment (estimate = 0.15, 95% *CI* = 0.09, 0.22), which provided support for H4.

**Table 2:** Results of the path analysis of the mediating effect.

**Path**	**Estimate**	**SE**	**Lower and upper 95% CI limits**
**Test of direct relationships**			
Perceived support for innovation → deviant innovation behavior	0.18^***^	0.06	(0.06, 0.31)
Perceived support for innovation → innovation commitment	0.32^***^	0.04	(0.22, 0.43)
Innovation commitment → deviant innovation behavior	0.47^***^	0.06	(0.34, 0.60)
**Test of indirect relationships**			
Perceived support for innovation → innovation commitment → deviant innovation behavior (bootstrap)	0.15^***^	0.03	(0.09, 0.22)

*N = 393*,

*** *p < 0.001*.

**Figure 2:**
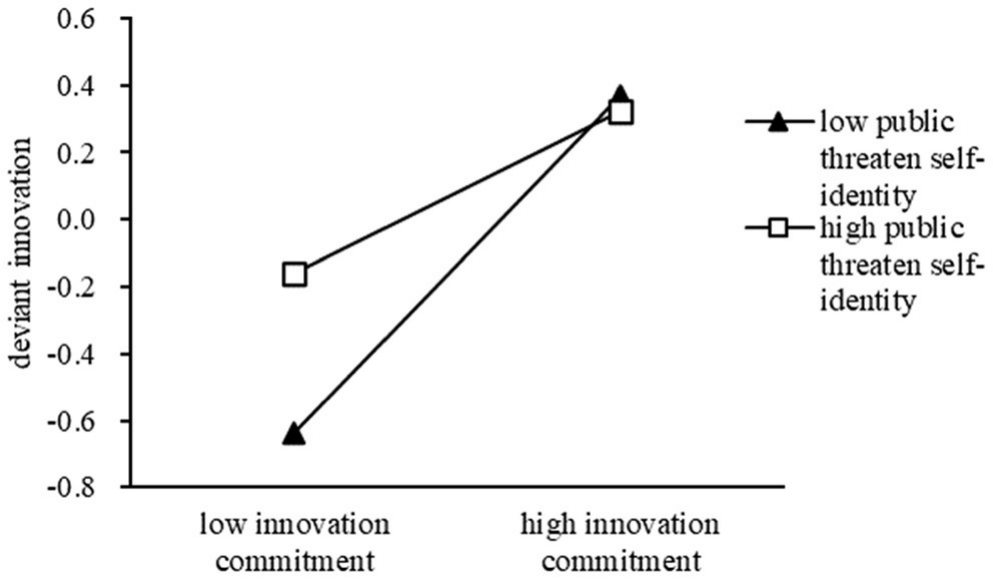
Moderating effect of public threat to self-identity on the relationship between innovation commitment and deviant innovation behavior.

Furthermore, we suggest that possible moderators should be considered to explain deviant innovation behavior. Thus, we proceeded to test for moderated mediation. H5 predicted that the effect of innovation commitment and deviant innovation behavior was moderated by threatened self-identity. The modeling results indicated a negative moderation effect of public threat to self-identity on the random slope between innovation commitment and deviant innovation behavior (γ = −0.13, *p* < 0.01). However, we found that the negative moderation effect of private threat to self-identity on the random slope between innovation commitment and deviant innovation behavior was not significant (γ = −0.08, *p*>0.05). Therefore, the form of the interaction was partially in the hypothesized direction. Additionally, to better comprehend the moderation of public threat to self-identity, we plotted the effect in Figure 2 (Aiken et al., 1991).

The results indicated that the indirect effect of perceived support for innovation on deviant innovation behavior via innovation commitment differed as a function of public threat to self-identity. That is, the negative indirect effect was weaker when there was a greater public threat to self-identity (estimate = 0.10, *SE* = 0.04, *p* < 0.05) and stronger when this threat was lower (estimate = 0.21, *SE* = 0.04, *p* < 0.05). Additionally, the difference in the indirect effects between the function of high and low levels of public threats to self-identity was significant (estimate = −0.13, *SE* = 0.05, *p* < 0.01). According to Hayes (2015), the index of moderated mediation was significant (estimate = −0.05, *SE* = 0.02, 95% *CI* = −0.10, −0.01), providing partial support for H6. In summary, the results from our path analysis provided strong support for our hypothesized process of the moderating effect of public threat to self-identity.

## Conclusion

The purpose of this study is to understand whether and how perceived support for innovation as an important individual difference affects deviant innovation behavior. Our results demonstrate that perceived support for innovation fosters deviant innovation behavior fully through innovation commitment and that public threat to self-identity buffers the positive effects of innovation commitment. The results of the analysis of data from 393 knowledge workers by SEM supported the hypotheses.

First, the results showed that perceived support for innovation has a positive direct effect on deviant innovation behavior. Because of the strong support for innovation from enterprises, knowledge workers satisfy their needs for efficacy, sense of power, and belonging and stimulate their rewards and reciprocal motives to the organization (Pierce et al., 2020; Wang and Yu, 2022; Xue, 2022). Possessions are often seen as extensions of self-awareness (Belk, 1988). Knowledge workers make the organization better by making more efforts to devise all types of creative solutions. Second, we also proved the mediation effect of innovation commitment. In China, people adopt the principles of both fairness and renqing when engaging in social exchanges (Ma et al., 2017; Ling et al., 2019). Perceived support for innovation is internalized as the motivation to reward the organization, leading to job involvement and more and higher-quality creative behavior (Gu et al., 2014a). Finally, we found that public threat to self-identity buffered the positive relationship between innovation commitment and deviant innovation behavior, as well as the indirect relationship between perceived support for innovation and deviant innovation behavior through innovation commitment. The bootlegging behavior is essentially about self-initiative (Nanyangwe et al., 2021). When knowledge workers take an active and self-starting approach to work and go beyond what is formally required in the given job, identification has been recognized as important for their deviant innovation behavior (Blader et al., 2017). And consciousness of social face is an important personal factor in China (Oetzel, 2008; Zhao and Bao, 2011). It is negatively correlated with interpersonal satisfaction and collaboration strategy (Liang and Duan, 2018). Public threat to self-identity can influence employees' in-role behavior in a safe direction.

Taking into account the results of the study, this research has theoretical and practical implications. In the case of theoretical implications, this study takes knowledge workers as the research object to explore the antecedent variables of deviant innovation behavior. It reveals the influential mechanism of perceived support for innovation on the deviant innovation behavior under the background of Chinese culture and verifies the mediating role of innovation commitment. This study also proves the moderating effect of public threat to self-identity, exposes the boundary conditions under which the perceived support for innovation influences the deviant innovation behavior of knowledge workers, and highlights the important role of situational factors on the whole mechanism. Previous studies have mostly examined the moderating or mediating effects of perceived support for innovation (Huang et al., 2016; Bosselut et al., 2020). Our findings highlight the influence of such perceived support on how knowledge workers treat their roles and the choice of innovation mode. Because knowledge workers often face complex and uncertain work conditions (Pearce, 2004), support from the organization can provide a sense of security. In addition, because of knowledge workers' work contains high creativity and autonomy, they often encounter problems of identity conflict and balance in terms of self-worth and self-efficacy under the influence of emotional events. Our findings are important for research because relationships with and comments by other people are more valued and play a particularly critical role in deviant innovation behavior in China (Fujiwara et al., 2016).

In the case of practical implications, managers should be fully aware of approaches to stimulating employee creative behavior. According to the findings of this paper, organizations should pay close attention to how employees perceive support for innovation. In line with previous studies (Gu et al., 2014b; Xu et al., 2021), we believe that perceptions of strong support for innovation can lead to positive outcomes. Although deviant innovation behavior has some risk and uncertainty, it is a spontaneous behavior and is good for organizations in essence (Wang, 2019). Managers should increase the confidence of knowledge workers and provide clear goals in various ways to fulfill their commitment to innovation. Organizations should optimize the institutional design to ensure full freedom and security. Furthermore, the psychological demands of these employees should be considered because feedback from the surroundings influences the motivation of the behavior. Organizations must alleviate employees' pressure at work, listen to their opinions and ideas, encourage them to view the value of innovation and properly address suggestions from others. In this way, organizations should improve the mechanisms of creative communication to successfully promote creative behavior. Moreover, managers should expand greater efforts to assist in the development of innovation commitment, which is helpful for achieving higher levels of innovation. Emotional events experienced by employees should also be given close attention in the workplace (Broekhuizen et al., 2017). When there is innovation failure or high pressure for innovation, entrepreneurs should create a strong democratic atmosphere and encourage employees to express their inner thoughts or dissatisfaction to promote a harmonious relationship and self- congruity.

The present research also has several limitations. First, although the data were collected at two stages, it would be better to measure perceived support for innovation and innovation commitment at two different times rather than at the same time. Second, we proposed only threats to self-identity, one of which, public threat to self-identity, buffers the positive indirect effect of perceived support for innovation on deviant innovation behavior through innovation commitment. Future studies should explore the buffering effects, which are not only from individual factors but also from organizational culture or situational characteristics such as person-job fit. Finally, the conclusion that public threat to self-identity buffered the positive indirect effect of perceived support for innovation on deviant innovation behavior through innovation commitment needs to be further tested in other populations and countries.

## Data Availability Statement

The original contributions presented in the study are included in the article/supplementary material, further inquiries can be directed to the corresponding author.

## Author Contributions

SY and XL designed the research and carried the investigation. XL analyzed the research data. SY wrote the manuscript. All authors contributed to the article and approved the submitted version.

## Funding

This work was supported by the Foundation for Outstanding Young Talents in College of Anhui Province (Grant No. gxyqZD2020103), the Key Project in Humanities and Social Sciences of Anhui Universities (Grant No. SK2021A0637) and the Philosophy and Social Science Planning Project of Anhui Province (Grant No. AHSKY2021D48).

## Conflict of Interest

The authors declare that the research was conducted in the absence of any commercial or financial relationships that could be construed as a potential conflict of interest.

## Publisher's Note

All claims expressed in this article are solely those of the authors and do not necessarily represent those of their affiliated organizations, or those of the publisher, the editors and the reviewers. Any product that may be evaluated in this article, or claim that may be made by its manufacturer, is not guaranteed or endorsed by the publisher.
